# Book Review

**Published:** 1940

**Authors:** 


### Reviews of Books

**Tuberculosis of Bone and Joint.** By G. R. GIRDLESTONE, F.R.C.S. Pp. xii., 265. Illustrated. London: Oxford University Press (Humphrey Milford). 1940. Price 30s.—The author of this book has been very fortunate both in his training and in the opportunities which he has enjoyed. One of Robert Jones's most able and enthusiastic pupils and colleagues, he was associated with that great man during the stirring days of the Great War, and was one of the pioneers of modern orthopædic surgery in this country. During the past twenty years he has been enabled to replace this by a modern open-air school hospital, which has been ever in the van of orthopædic progress. The present volume represents the author's considered experience and opinion on all the problems associated with tuberculosis of the bones and joints. It is very clearly written and well illustrated. The chapters on the hip and spine give a very complete account of the pathology, treatment and prognosis of tuberculous disease of these structures. The book does not profess to give a complete account of various opinions and practices, but consists merely of a description of the author's own practice, with some indications of the results obtained. Many authorities will not agree with all his advice and conclusions, but the book will remain as a faithful exposition of the practice of a very earnest student of the subject. The chief point of criticism which will be levelled against Mr. Girdlestone is that whilst he preaches the doctrine of conservatism he advises the practice of operative interference under conditions which are open to considerable doubt.

**An Introduction to Medical Genetics.** By J. A. F. ROBERTS, M.B., D.Sc., F.R.S.E. Pp. xxiii., 266. Illustrated. London: Oxford University Press (Humphrey Milford). 1940. Price 15s.—To many people medical genetics is an unknown world vaguely associated with genes and higher mathematics. Actually the subject is intimately connected with ordinary clinical work and the contributions of geneticists during the last thirty years have thrown invaluable light on many obscure problems. Many questions that could only be answered with a well-intentioned guess can now be dealt with accurately. Dr. Fraser Roberts' book presents the whole subject in extremely readable form. It is probably too full for the already overworked medical student, but should prove invaluable for all those working for higher examinations. The book is excellently produced and is up to the high standard that is always attained by the Oxford University Press, and should stimulate many people to take a more intelligent interest in the whole subject of heredity.

**Practical Food Inspection.** Second Edition. By C. R. A. MARTIN, M.R.San.I. Vol. I. : pp. viii., 316, price 15s. Vol. II. : pp. viii., 275, price 12s. 6d. Illustrated. London : H. K. Lewis & Co. Ltd. 1940. —A second edition of these two volumes appearing so soon indicates the need for a work of this character. The author appears to have covered every possible aspect of the practice of food inspection and has presented his conclusions in an informative and easily readable manner. Legislation dealing with the practice is couched in simple and concise phrases. The new procedure under the Food and Drugs Act, 1938, brings it right up to date. The work is generously illustrated. The original sketches are excellently drawn and so clearly intelligible as to give a very good idea of the characteristics of the diseases found in food animals. Food inspectors generally have appreciated the first edition and regard them as text-books. The new edition therefore, being enlarged with the most recent information, will be welcomed by these and by the student preparing to qualify as a meat and food inspector. Medical Officers of Health and Veterinary Officers will find the extensive practical experience of the author a very useful aid. The index is full and complete, the general format excellent, and it can be fully recommended as a well-written practical exposition of food inspection. The great value of this work is that within its covers are contained all the essential principles of this comprehensive subject tersely and lucidly described.

**A Text-Book of Psychiatry.** Fifth Edition. By D. K. HENDERSON, M.D., F.R.F.P.S., F.R.C.P.E., and R. D. GILLESPIE, M.D., F.R.C.P., D.P.M. Pp. xii., 660. London : Oxford University Press (Humphrey Milford). 1940. Price 20s.—The fact that five editions of this work have been published since 1927 is evidence of its popularity. The present edition follows the general form of its predecessors, with the addition of chapters on play therapy and child guidance work and on modern shock treatment. The views expressed on both subjects are cautious but encouraging, and the whole book can be confidently recommended to all those who are interested in the subject.

**Medical Annual, 1940.** Edited by H. LETHEBY TIDY, D.M., F.R.C.P., and A. RENDLE SHORT, M.D., F.R.C.S. Pp. 604. With 62 figures and 65 plates. Bristol : John Wright & Sons Ltd. Price 20s.—Wright's *Medical Annual* for 1940 continues to offer an all-round survey of the progress of medicine in all its branches. The role of sulphonamides in therapeutics is discussed in various articles. In surgery the articles on war injuries in the light of Spanish war experiences will be read with particular interest. An article on the medical hazards of submarine crews has a particular freshness and importance. That widespread nuisance "Athlete's Foot" or interdigital ringworm of the toes is dealt with in a short and valuable contribution. Industrial medicine is given more attention than usual in this volume, but not more than it demands in these times of high pressure work in factories. It is hard to find anything new that goes unnoticed in our old friend the *Medical Annual* ; but there is a great advantage in having novelties critically appraised by workers of experience, as they are in this compendium.

